# Study protocol of a phase II clinical trial evaluating the efficacy of neoadjuvant intraperitoneal and systemic albumin-bound paclitaxel combined with camrelizumab and S-1 in the treatment of patients with exfoliative cell-positive gastric cancer

**DOI:** 10.3389/fonc.2023.1201928

**Published:** 2023-09-29

**Authors:** Jingxia Lv, Jiaxiang Wu, Haotian Wu, Ping’an Ding, Honghai Guo, Peigang Yang, Yuan Tian, Yang Liu, Qun Zhao

**Affiliations:** ^1^ The Third Department of Surgery, the Fourth Hospital of Hebei Medical University, Shijiazhuang, China; ^2^ Hebei Key Laboratory of Precision Diagnosis and Comprehensive Treatment of Gastric Cancer, Shijiazhuang, China

**Keywords:** neoadjuvant intraperitoneal and systemic, conversion treatment, gastric cancer, positive lavage cytology, camrelizumab

## Abstract

**Background:**

Currently, gastric cancer with positive lavage cytology without gross peritoneal dissemination (GC-CY1) is a special type of metastatic form with poor prognosis. Consensus guidelines on treatment strategies for patients with GC-CY1 have not been established. This study involves a single-arm, prospective, phase II clinical trial to examine the efficacy and safety of neoadjuvant intraperitoneal and systemic (NIPS) albumin-bound paclitaxel combined with Camrelizumab and S-1 in the treatment of GC-CY1 patients.

**Methods/design:**

This is a prospective single-center exploratory study, and the primary endpoints of the trial are R0 resection rate and conversion rate of abdominal free cancer cells (FCCs), with secondary endpoints of 3-year progression-free survival (PFS); 3-year overall survival (OS); objective remission rate (ORR); disease control rate (DCR); safety and TRG classification.

**Discussion:**

This study is the first to apply NIPS albumin-bound paclitaxel combined with Camrelizumab and S-1 to the conversion therapy of GC-CY1 patients. It is speculated that this combination of regimens will increase the negative conversion rate of FCCs by 20%, which will provide innovative insights into conversion treatment ideas for GC-CY1 patients to be managed in a more comprehensive and optimized manner.

**Clinical trial registration:**

http://clinicaltrials.gov/, identifier NCT05410847.

## Background

Gastric cancer is the fourth most common malignancy worldwide, with the third highest mortality rate (1, 2). Presently, peritoneal metastasis is a common form of metastasis in advanced gastric cancer, and positive free cancer cells (FCCs) in the abdominal cavity is one of the special ways (3). Currently, gastric cancer with positive lavage cytology without gross peritoneal dissemination (GC-CY1) is categorized as M1 and classified as stage IV in both the UICC TNM 8th editions (4) and the Japanese classification of gastric carcinoma 3rd English edition (Japanese classification) (5). Numerous studies have found that the detection of FCCs in intraoperative peritoneal fluid is an important risk factor affecting the prognosis of patients, and the median survival time is 12.5-13.8 months, which was unsatisfactory (6–8).

In order to improve the long-term prognosis of GC-CY1 patients, multiple treatment modalities have recently received increasing attention. Till this moment, there is no unified consensus on the treatment strategy for GC-CY1 patients. The REGATTA trial (9), a phase III study that included 175 patients with advanced gastric cancer with a single incurable factor (including liver metastasis, peritoneal metastasis or para-aortic lymph node metastasis) found that chemotherapy after gastrectomy did not show any survival benefit compared with chemotherapy alone (16.6 months vs 14.3 months, HR=1.09, 95%CI: 0.78-1.52). Therefore, direct gastrectomy is not applicable for such patients, and conversion therapy may become the next strategy for stage IV gastric cancer. A retrospective study that included 100 patients with GC-CY1 investigated whether radical gastrectomy and lymph node dissection improved patient prognosis, but found that the median survival time for patients who underwent gastrectomy was 21.7 months compared with 20.5 months for patients who were not treated with primary surgery (p = 0.155) (10). This study suggests that gastrectomy is not effective in improving survival time in patients with GC-CY1 and that chemotherapy should be prioritized as initial treatment. Meanwhile, Yamaguchi et al. conducted a multicenter retrospective study on whether preoperative chemotherapy could be used to improve the efficacy of initial treatment in patients with GC-CY1 (11). A total of 713 eligible patients were enrolled in this study, 150 of whom received chemotherapy as initial treatment and 563 of whom underwent surgery, with similar overall survival (OS) (median OS 24.8 vs. 24.0 months, HR=1.07, 95%CI: 0.87-1.30)and progression-free survival (PFS) (median PFS 14.9 vs. 13.9 months, HR=1.04, 95%CI: 0.85-1.27) in both groups. This study suggests that while preoperative chemotherapy did not show a survival benefit in patients with GC-CY1, initial chemotherapy showed favorable survival in patients with successful conversion.

However, numerous studies have also demonstrated that systemic chemotherapy has little efficacy in GC-CY1 patients because of the presence of the plasma-peritoneal barrier, which is difficult for larger molecular chemotherapeutic agents to cross and act on microscopic metastases in the abdominal cavity (12, 13). In recent years, neoadjuvant intraperitoneal and systemic (NIPS) have attracted more and more attention, which can be applied directly to the abdominal cavity and kill tumor cells through contact between chemotherapeutic drugs and FCCs in the abdominal cavity (14–17). PHOENIX-GC, a multicenter prospective randomized controlled study, is the first to use NIPS paclitaxel in GC-CY1 patients, and although its results were negative it opens up new ideas for the conversion therapy of GC-CY1 patients, which is encouraging (18). We have previously carried out a single-arm prospective study of conversion therapy in GC-CY1 patients, in which we used a preoperative 3-cycle regimen of NIPS paclitaxel in combination with apatinib and S-1, with a 77.78% conversion rate of FCCs (19). This study suggests to us that the optimal selection of chemotherapeutic agents under the intraperitoneal and intravenous routes is critical to the success of treatment, and that the choice of targeted or immunotherapeutic agents based on the expression of tissue markers is also an important factor in the efficacy of treatment. In conclusion, it has also been identified that albumin-bound paclitaxel may have better activity and higher intraperitoneal drug concentration compared to paclitaxel injection (20–22). Consequently, in this study, we used albumin-bound paclitaxel for the first time in the treatment of NIPS to observe the success rate of translational therapy of FCCs.

Currently, immunotherapy is also attracting increasing attention worldwide, and a series of prospective studies have been performed on neoadjuvant chemotherapy for locally advanced gastric cancer, with more satisfactory results so far, including the CheckMate 649 trial (23), the ATTRACTION-4 trial (24), and the KEYNOTE-811 trial (25). Meanwhile, in addition to its application during neoadjuvant chemotherapy for locally advanced gastric cancer, immunotherapy has also been gradually applied to the conversion treatment of distant metastatic gastric cancer (26, 27). Camrelizumab (Jiangsu Hengrui Pharmaceuticals Co, Ltd), a humanized, selective IgG4-κ monoclonal antibody against PD-1, exerted antitumor activity in a wide range of tumors (28–32). In multiple prospective studies, camrelizumab significantly improved overall survival and remission rates compared to chemotherapy as second-line therapy for patients with advanced or metastatic gastric cancer (27, 33). The combination of immunotherapy and cytotoxic agents has shown satisfactory antitumor activity in a variety of gastrointestinal malignancy types (34, 35), but there is a lack of evidence to support whether this combination regimen can also be used as a first-line translational treatment strategy for patients with GC-CY1.

In this context, we selected GC-CY1 patients for this exploratory prospective clinical trial (FUTURE-02 study) with a regimen of NIPS albumin-bound paclitaxel combined with Camrelizumab and S-1 to evaluate the conversion therapeutic efficacy and adverse events of this combined regimen in untreated GC-CY1 patients.

## Methods/design

### Study setting

**Figure 1:**
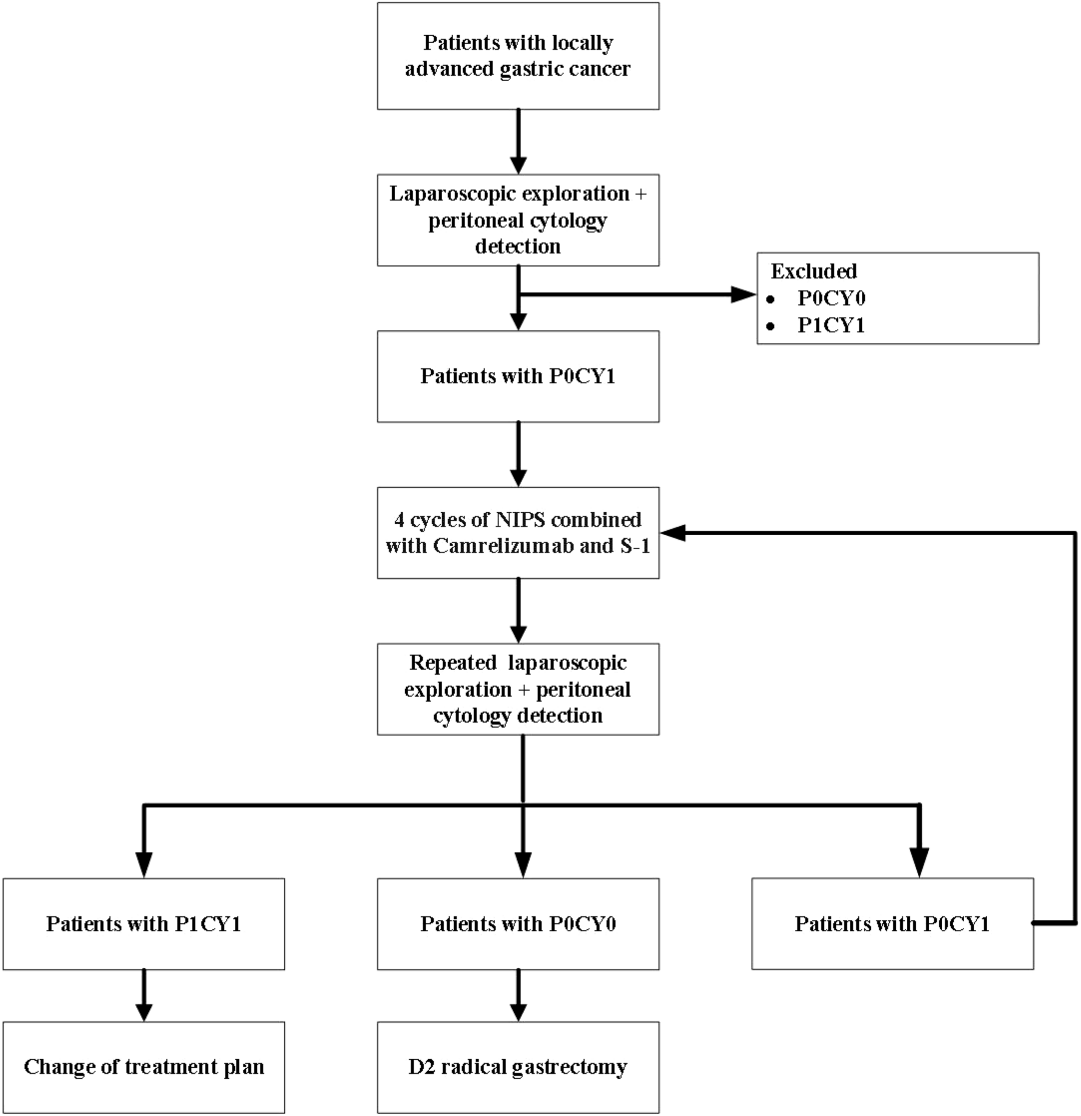
Study Flow Chart.

FUTURE-02 is a single-center prospective exploratory trial. Eligible patients with locally advanced gastric cancer underwent laparoscopy and peritoneal exfoliative cytology, and GC-CY1 patients were selected for inclusion in the trial. All enrolled GC-CY1 patients received 4 cycles of neoadjuvant intraperitoneal and systemic (NIPS) albumin-bound paclitaxel combined with Camrelizumab and S-1, and then underwent laparoscopic exploration and peritoneal cytology again. If the FCCs turned negative, radical resection was performed, and if it was still positive, the conversion treatment of the original regimen was continued (**Figure 1**). This study was approved by the Ethics Committee of the Fourth Hospital of Hebei Medical University (approval number: 2021117) and registered with the ClinicalTrials.gov under the registration number NCT05410847. All patients entering the study need to sign informed consent. Monitoring will be carried out throughout the test.

### Endpoints

The primary endpoint for the current trial will be R0 resection rate and conversion rate of abdominal FCCs, with secondary endpoints of 3-year progression-free survival (PFS); 3-year overall survival (OS); objective remission rate (ORR); disease control rate (DCR); safety and TRG classification.

Pathologic response was evaluated and graded according to the TRG classification criteria (AJCC/CAP criteria) (36). TRG 0 is defined as no residual tumour cells found microscopically on multiple consecutive sections. If there was an ulcer on the surface of the lesion, and the small clusters of tumor cells only presented in the subserous layer, then it was defined as TRG 1. Fragmented residual tumor cells with fibrosis and inflammatory cells in the lesion were defined as TRG 2. If there was almost no fibrosis in the lesion, then the tumor cells were defined as TRG 3. Four weeks after completing conversion treatment, tumor resectability and objective efficiency were assessed by CT. The ratio of TRG 0 to TRG 1 in all patients was defined as the main pathological response (MPR), and the tumor regression grade except TRG 3 was defined as pathological response rate (pRR).

Tumor response was evaluated in accordance with the Response Evaluation Criteria in Solid Tumors (RECIST) 1.1 (37), which was divided into complete response (CR), partial response (PR), stable disease (SD) and progressive disease (PD). CR and PR cases were defined as effective chemotherapy and their percentage in all patients was considered ORR. Moreover, CR, PR, and SD were considered disease control and their percentage among all patients was regarded as DCR.

During the conversion therapy, the National Cancer Institute Common Terminology Criteria for Adverse Events (version 4.0) (38) were used to evaluate the classification of adverse reactions. AEs were recorded by investigators, and the relationship between AEs and treatment was assessed.

### Patient recruitment and sample size estimation

To the best of our knowledge, there are no studies evaluating the relationship between NIPS albumin-bound paclitaxel combined with Camrelizumab and S-1 and the efficacy of conversion therapy in GC-CY1 patients; therefore, it is not possible to estimate the optimal sample size by efficacy calculations.

In this prospective exploratory study, we aimed to include 30 patients with GC-CY1, who were recruited from The Third Department of Surgery of the Fourth Hospital of Hebei Medical University. Approximately 280 new patients present to the department each year, and of those who undergo laparoscopic exploration and abdominal FCCs testing, 14.22% are subsequently diagnosed with GC-CY1 (39). These figures suggest that our projected goal of recruiting 30 people within 24 months is achievable.

### Eligibility criteria and exclusion criteria

#### Inclusion criteria:

1. Patients who have not received chemotherapy, radiotherapy or other anti-tumor treatments before the start of the clinical trial;
2. Aged between 18 and 70 years old;
3. Men or non-pregnant or lactating women;
4. Gastric adenocarcinoma was diagnosed by gastroscopy and pathology, and Human Epidermal GrowthFactor Receptor 2 (HER-2) was negative by immunohistochemistry;
5. Imaging examination confirmed that the T stage was T3 or T4, and there was no macroscopic distant metastasis during the operation; the exfoliated cytology of peritoneal lavage fluid was positive;
6. Blood routine meets the following conditions: white blood cell count ≥ 3.5×10^9^/L, neutrophil ≥ 1.5×10^9^/L, platelet count ≥ 100×10^9^/L, hemoglobin ≥ 90 g/L;
7. Biochemical tests meet the following conditions: ALT ≤2.5×Upper Limit of Normal (ULN), AST ≤2.5×ULN, Serum Total Bilirubin ≤1.5×ULN, Serum Creatinine ≤1.5×ULN;
8. Left ventricular ejection fraction ≥ 50%;
9. Eastern Cooperative Oncology Group (ECOG) score≤ 2;
10. Able to abide by the protocol during the study period and voluntarily sign the informed consent form.

#### Exclusion criteria:

1. Immunosuppressive drugs have been used within 14 days before taking camrelizumab, excluding nasal spray and inhaled corticosteroids or systemic steroids at physiological doses (ie, no more than 10 mg/day prednisolone or other corticosteroids at equivalent physiological doses);
2. History of any active autoimmune disease or autoimmune disorders while using camrelizumab, including those of the digestive system (enterocolitis, hepatitis), endocrine system (hyperthyroidism, hypothyroidism), respiratory system (interstitial pneumonia, asthma), and other systemic diseases (vasculitis, vitiligo, uveitis);
3. History of other pathologically confirmed malignancies within 5 years of the diagnosis of gastric cancer;
4.Human immunodeficiency virus (HIV) infection or known acquired immunodeficiency syndrome (AIDS), active hepatitis B (HBV DNA ≥ 1000 IU/ml), hepatitis C (positive hepatitis C antibody, and HCV- RNA is higher than the detection limit of the analysis method) or co-infected with hepatitis B and C, and patients who need antiviral treatment during the study;
5. Other transferred organs;
6. Patients with serious or uncontrolled medical diseases and infections (including atrial fibrillation, angina pectoris, cardiac insufficiency, ejection fraction lower than 50%, uncontrolled hypertension, etc.);
7. Those who have a history of psychotropic drug abuse and cannot quit or patients with mental disorders;
8. Patients with severe or uncontrollable mental illness;
9. According to the investigator’s judgment, patients with concomitant diseases that seriously endanger the safety of patients or affect the completion of the study;
10. The researchers think that they are not suitable for inclusion.

### Chemotherapy regimen

**Figure 2:**
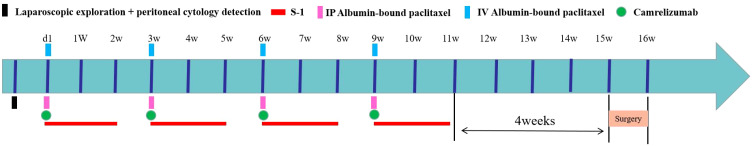
Chemotherapy regimen.

All enrolled patients underwent implantation of peritoneal chemotherapy ports using the methodology employed in our previous studies (19). Prior to inclusion, all patients were subjected to diagnostic laparoscopy and intra-abdominal free cancer cell testing. Patients included in the first clinical trial started treatment on the next day of laparoscopic exploration, and each treatment cycle lasted for 3 weeks. On day 1 of the treatment cycle, albumin paclitaxel was infused via an IP chemotherapy pump (IP route 80 mg/m^2^ over 1 hour) and intravenously (IV route 180 mg/m^2^ over 1 hour). At the meantime, Carrilizumab 200 mg/dose was given by the IV route (IV drip for 30 min, not less than 20 min and not more than 60 min), plus 14 days of continuous oral S-1 after the completion of the infusion (**Figure 2**).

The oral dose of S-1 in all GC-CY_1_ patients included in these two clinical trials was calculated according to the body surface area (BSA), as follows: for BSA <1.25 m^2^, 80 mg/(m^2^·d) S-1 was administered; for BSA 1.25-1.50 m^2^, 100 mg/(m^2^·d) S-1 was administered; and for BSA >1.50 m^2^, 120 mg/(m^2^·d) S-1 was given. Oral S-1 (a contemporary oral fluoropyrimidine) was given 30 minutes after breakfast and 30 minutes after dinner for 14 consecutive days.

All patients underwent laparoscopic exploration and peritoneal cytology test after 4 cycles of conversion therapy, and the treatment plan was determined according to the intraoperative exploration. If FCCs turned negative and no visible peritoneal metastasis was found, then the radical resection was performed. If FCCs were still positive but no peritoneal metastasis occurred, the conversion therapy was continued according to the original scheme; if peritoneal metastasis occurred in abdominal exploration, the conversion therapy was replaced.

### Follow-up

All patients were recommended to have a follow-up visit every 3 months in the first 2 years, and every 6 months after 2 years. Follow-up methods mainly included telephone encounters, outpatient visits, and hospitalizations. Examinations to be done on admission included CT of the chest, abdomen, and pelvis, esophagogastroduodenoscopy (EGD) and blood tests for tumor markers (carbohydrate antigen 199 [CA19-9], CA72-4, carcinoembryonic antigen [CEA], and alpha-fetoprotein [AFP]). OS was defined as the time interval from treatment to cancer-related death or final follow-up visit, and OS was the preferred destination. And PFS was measured from the time of treatment initiation to clinical or radiographic progression or death from any cause.

### Statistical analysis

Statistical analysis was performed using SPSS 21.0. Descriptive data were presented as mean ± standard deviation, while categorical data were expressed as numbers and percentages (%). Independent samples t-test or nonparametric tests were utilized to analyze continuous data for metric variables, while the chi-square test or Fisher’s exact test was employed for analyzing categorical variables in count data. Kaplan-Meier method was employed for survival curve plotting, and single-factor survival analysis was conducted using the Log-rank test, while multivariate survival analysis was carried out using the Cox regression model. A significance level of P<0.05 was considered indicative of statistically significant differences.

## Discussion

Numerous studies have found that patients with gastric cancer presenting with peritoneal metastases at the time of initial diagnosis are difficult to resect surgically and have an extremely poor prognosis, with a median survival time of only 6 to 9 months. In recent years, GC-CY1 patients as a special metastatic type of gastric cancer, the selection of its optimal treatment plan has been of great concern. Numerous clinical practice results have shown that for GC-CY1 patients, the effect of preoperative NIPS chemotherapy is significantly better than that of traditional systemic chemotherapy alone. This study was performed to investigate the feasibility and efficacy of more aggressive improvement in the conversion rate of FCCs in GC-CY1 patients receiving conversion therapy. This proposed study investigates a more comprehensive approach to the management of GC-CY1 patients on preoperative conversion therapy, in which it is hypothesized that NIPS albumin paclitaxel may provide local management of occult peritoneal metastases or FCCs, and that systemic intravenous administration of albumin paclitaxel as well as carrelixumab and S-1 may induce tumor shrinkage.

Due to the presence of the peritoneal plasma barrier in the human body, the concentration of chemotherapeutic drugs within the peritoneal tissues is often quite low when administered systemically (12, 13). This barrier presents a specific challenge for large molecular drugs, as their capacity to traverse this barrier is restricted, consequently impeding the effectiveness of chemotherapy (40). However, with intraperitoneal administration, the peritoneal plasma barrier assumes a proactive role by slowing down the absorption rate of drugs by the peritoneum. Consequently, drugs remain within the peritoneal cavity for an extended duration, leading to the establishment of higher drug concentrations.

Within such a drug-rich environment, medications exert their effects on the surface of tumor metastases, maintaining an extended duration of action, thus facilitating therapeutic outcomes. In this research endeavor, we have adopted a bidirectional treatment approach known as NIPS, where a combination of systemic chemotherapy (via intravenous and oral routes) is employed alongside intraperitoneal drug administration. Through systemic chemotherapy, drugs are transported via blood vessels to the inner regions of tumor tissues, manifesting their therapeutic effects therein. Concurrently, intraperitoneal administration allows drugs to act directly on the surface of tumor metastases (19).

Theoretically, this dual approach enables drug action both on the tumor surface and within its interior, thereby achieving a dual therapeutic effect on tumor metastases. It is important to note, however, that while this strategy has the potential to significantly increase drug concentration at tumor sites, it may also elevate the risk of drug metabolism and adverse effects. Consequently, further assessments of safety and efficacy are imperative in the context of clinical application.

However, this strategy also presents certain limitations. Firstly, there is currently no established consensus on the sequential treatment regimen for the NIPS therapy. In this prospective study, we have defined the conversion therapy cycle as four cycles based on the preliminary research results. In future investigations, we plan to incorporate dynamic monitoring of tumor residual status using minimal residual disease (MRD) techniques during the post-treatment interval, which will guide further intraperitoneal exploration and assessment of conversion therapy outcomes (41). Secondly, there lacks a unified perspective on the postoperative adjuvant treatment regimen and duration for successfully converted GC-CY1 patients. The post-treatment management and follow-up after completing the therapeutic course within the study protocol are pivotal. It is well recognized that the enhancement of conversion therapy success rate serves as a short-term observatory index, whereas the long-term prognosis of GC-CY1 patients holds paramount clinical significance. Therefore, our forthcoming research endeavors will place significant emphasis on the selection of treatment approaches and prognostic analyses for these patients following conversion therapy.

This is indeed the first prospective exploratory study that will investigate the combined translational therapeutic role of NIPS albumin paclitaxel in combination with carrelicizumab and S-1 in the treatment of patients with GC-CY1. The results of this study will help set the standard of care for clinical practice in GC-CY1 patients with FCCs.

## Data availability statement

The original contributions presented in the study are included in the article/**Supplementary Material**. Further inquiries can be directed to the corresponding author.

## Ethics statement

The studies involving humans were approved by the Ethics Committee of the Fourth Hospital of Hebei Medical University (approval number: 2021117) and registered with the ClinicalTrials.gov under the registration number NCT05410847. All patients entering the study need to sign informed consent. Monitoring will be carried out throughout the test. The studies were conducted in accordance with the local legislation and institutional requirements. Written informed consent for participation was not required from the participants or the participants’ legal guardians/next of kin in accordance with the national legislation and institutional requirements. The manuscript presents research on animals that do not require ethical approval for their study.

## Author contributions

(I) Conception and design: QZ. (II) Administrative support: QZ (III) Provision of study materials or patients: JL, P’AD, PY, YT, HG. (IV) Collection and assembly of data: JL, P’AD, PY, YT, HG. (V) Data analysis and interpretation: P’AD, HG, HW, JW. (VI) All authors contributed to the article and approved the submitted version.
