# Supplementary material for: Study protocol of a phase II clinical trial evaluating the efficacy of neoadjuvant intraperitoneal and systemic albumin-bound paclitaxel combined with camrelizumab and S-1 in the treatment of patients with exfoliative cell-positive gastric cancer
Source: Front Oncol. 2023 Sep 29;13:1201928. doi: 10.3389/fonc.2023.1201928 (PMC10571916; doi:10.3389/fonc.2023.1201928)
Supplement: Supplementary file 1 [file DataSheet_1.pdf]

A prospective clinical study of NIPS combined with carlizumab and tigio transformation in exfoliated cytologically positive gastric cancer

Version number: 1.0

Version date: 20217.20.0

Project leader: Zhao Qun

Department: The Fourth Hospital of Hebei Medical University

Project execution time: July 2021 to July 2025

catalogue

Protocol summary **4**

1. research background **8**

2. Study objectives, content, and significance **13**

3. implementation plans **14**

research design 14

technology roadmap 15

Implementation steps 16

(I) Inclusion criteria 16

(3) Exit criteria 17

(4) Elimination criteria 17

(V) Screening and enrollment stage 17

(VI) Post-enrollment treatment plan 18

(Vii) Follow-up plan and content 20

(8) Treatment of tumor recurrence and metastasis 23

(IX) Ethical requirements and the informed consent form from the subjects 23

(10) Progress and completion time of clinical trials 23

(11) The preservation of the data 24

reference documentation **24**

Appendix 1 Standardized operation procedures for radical gastrectomy **27**

Adp. 2 Dose adjustment for toxicity **30**

Appendix 3 Tigio dose calculated based on body surface area **37**

Appendix 4, ECOG PS Score **38**

Appendix 5 Standardized operation procedure for postoperative intraperitoneal chemotherapy port placement for gastric cancer **39**

SCHEME SIGNATURE PAGE

I, as the doctor involved in the study, have read the protocol for the study.

I have fully discussed the purpose of the study and the content of the protocol.

I agree to conduct the study under this protocol, to comply with the ethics requirements, and to conduct this clinical study under the guidance of Good Clinical Practice (GCP).

I agree that the contents of the protocol will be kept confidential and will not be disclosed to third parties, and that the contents of the protocol will only be used for conducting this study.

I understand that if the decision to prematurely terminate or suspend it is made at any time and for any reason, I will be informed in writing. Similarly, if I decide to withdraw from performing the study, I will also immediately notify the study principal investigator in writing.

Name: _______________

Date: ________________

PROTOCOL SUMMARY

research topic		A prospective clinical study of NIPS combined with carlizumab and tigio transformation in exfoliated cytologically positive gastric cancer
version number		1.0
Bid unit		The Fourth Hospital of Hebei Medical University
Research nature		exploratory research
subject investigated		Patients with gastric cancer with positive shedding cytology
study purpose	fundamental purpose	R0 resection rate; negative turn rate
	Secondary purpose	3-year progression-free survival rate (PFS), 3-year overall survival rate (OS); objective response rate (ORR); disease control rate (DCR); safety; TRG classification;
Exploratory indicators		Association between biomarkers and efficacy prediction (including but not limited to PD-L1 expression, T cell subsets, etc.)
Planning of the number of enrolled patients		Thirty cases were planned to be enrolled
investigator		Zhao Qun
Research unit		The Fourth Hospital of Hebei Medical University
Patient screening criteria		Inclusion criteria:<br>1. Primary treatment patients, without chemotherapy, radiotherapy or other anti-tumor therapy before the start

of the clinical trial;

2. Age: 18-70 years old;
3. Male or non-pregnant or lactating women;
4. Gastroscopy and pathology confirmed gastric adenocarcinoma, and the immunohistochemical test was negative for HER-2;
5. Imaging examination confirmed T stage as T3 and T4, and no macroscopic distant metastasis; positive cytology;
6. The blood routine meets the following conditions: white blood cell count 3.510^9/ L, 1.510 for neutrophils^9/ L, platelet count of 10010^9/ L, hemoglobin of 90 g / L;
7. Biochemical examination meets the following conditions: ALT 2.5 upper limit of normal value (ULN), AST 2.5 ULN, serum total bilirubin 1.5 ULN, blood creatinine 1.5 ULN;
8. LV ejection fraction 50%;
9. ECOG score: 0-1;

10 Be able to comply with the protocol during the study period and voluntarily sign the informed consent form.

Exclusion criteria:

1. Use of immunosuppressive drugs within 14 days prior to the use of carlizumab, excluding nasal and inhaled corticosteroids or physiological doses of systemic steroid hormones (i. e., no more than 10 mg / day of prednisolone or other corticosteroids at equivalent physiological doses);
2. With carlizumab, there was any active autoimmune disease or autoimmune history (including but not limited to autoimmune hepatitis, pneumonia, interstitial pneumonia, uveitis, enteritis, hepatitis, pituitary, vasculitis, nephritis, hyperthyroidism, reduced thyroid function; vitiligo or asthma in childhood, adults

	without any intervention; asthma requiring medical intervention with bronchodilators could not be included);<br>3. History of other malignancies within 5 years;<br>. 4 patients with human immunodeficiency virus (HIV) infection or known acquired immunodeficiency syndrome (AIDS), active hepatitis B (HBV DNA 1000 IU / ml), hepatitis C (hepatitis C antibody positive and HCV-RNA above the lower limit of detection of the analytical method) or combined hepatitis B and hepatitis C coinfection requiring antiviral treatment during the study;<br>6. Other transferred organs;<br>7. Severe or uncontrolled medical diseases and infected persons (including atrial fibrillation, angina pectoris, cardiac insufficiency, ejection fraction less than 50%, difficult to control hypertension, etc.);<br>8. Patients with a history of psychiatric substance abuse and unable to quit or with mental disorders;<br>9. Severe or uncontrolled mental illness;<br>10. Patients with concomitant diseases that seriously endanger patient safety or affect the patient to complete the study according to the discretion of the investigator;<br>11.
End of the study standard of treatment	1. The patient has completed the treatment specified in the protocol;<br>2. Do not want to continue receiving treatment in the event of adverse events (AEs) or serious adverse events (SAEs);<br>3. Grade 3 / 4 adverse events could not be treated according to the trial protocol after dose adjustment;<br>4. Unanticipated and unacceptable adverse drug reactions

	occur;<br>5. Other circumstances that the investigator considers it necessary to withdraw from the study;
Apping / removal criteria	1. At the same time, other CFDA approved anti-gastric cancer chemotherapy drugs, immune checkpoint inhibitors;<br>2. Failure to use the dose, method and course of treatment specified in the study protocol;
Withdrawal from study criteria	1. Allergic reaction occurs<br>2. patient requested withdrawal
dosage regimen	Patients with gastric cancer confirmed as CY 1P 0 after laparoscopic exploration were included, and all patients received NIPS combined with carilizumab and tigiol, drug selection and dose:<br>Carrelizumab: 200m g / time, intravenous 30min infusion (not less than 20min and not more than 60min), q 3w. A total of 4 cycles.<br>NIPS with albumin paclitaxel: intraperitoneal dose of 80mg / m^2, D 1; intravenous dose of 180mg / m^2,d1. Every 21 days is one treatment cycle for 4 cycles.<br>Tigio: BSA <1.25m^2，40mg bid，1.25m^2≤BSA≤1.5m^2，50mg bid， BSA >1.5$m^{2\cdot}$60mg bid, the first dose was taken on the first night, and the last dose was taken in the morning on day 15 with intermittent chemotherapy.1 cycle every 3 weeks, 2 weeks per gigiintake, 1 week rest for 4 cycles.<br>In operable patients, the original regimen was continued for adjuvant therapy for 4 cycles, after which carelizumab monotherapy was maintained until 1 year.<br>Inoperable patients are recommended by the investigator

	according to the guidelines.
. Main efficacy indicators	R0 resection rate; negative turn rate
Secondary efficacy indicators	3-year progression-free survival rate (PFS), 3-year overall survival rate (OS); objective response rate (ORR); disease control rate (DCR); safety; TRG classification;
Primary safety indicators	Vital signs, laboratory indicators, adverse events (AEs), serious adverse events (SAEs)
Exploratory indicators	Association between biomarkers and efficacy prediction (including but not limited to PD-L1 expression, T cell subsets, etc.)

1. research background

.1 1 The treatment profile of gastric cancer

Despite the trend of gradual decline in the prevalence and mortality of gastric cancer, gastric cancer still has the second highest mortality rate worldwide[1-2]China is in the area of high incidence of gastric cancer, the annual incidence of gastric cancer is 23.7/10 ten thousand and the fatality rate is 16.6/10 million. Compared with Japan and South Korea, most gastric cancer patients in China are already in the advanced stage, while early gastric cancer accounts for less than 10%[3]. For advanced gastric cancer, the disease recurrence rate remains high in both countries or regions[4-7], Therefore, the diagnosis and treatment of advanced gastric cancer is of great significance in China. And D'Angelica et al[8]Considering peritoneal implant metastasis as the main prognostic factor for advanced gastric cancer, 496 (42.3%) of 1172 R0 resection gastric adenocarcinoma at Memorial SloanKettering Cancer Center (MSKCC) had tumor recurrence, in 48.8% of all recurrent patients.

It has been reported that recurrence is associated with the depth of tumor invasion, and about 30% -50% of gastric cancer patients involving the subserosal layer or the serosal layer already have abdominal free peritoneal cancer cells (FCC)[9-10]. D'Angelica et al reported that patients with gastric cancer involving the subserosal or serosal layer, regardless of the type of Lauren, tumor location, were still 5 times more likely for peritoneal recurrence than those with gastric cancer involving only the T0-2 layers[8]. Ikeguchi Reported that the serinfiltration area and intraperitoneal tumor cells were positively correlated, and the 5-year survival rate of negative free cancer cells was 49.3%, but patients with free cancer cells[11]. At present, the accuracy of T stage and N stage by common clinical imaging tests can reach 70% -80%, but the positive detection rate of these small disseminated metastases is low. Preoperative laparoscopic exploration can observe the site, range, depth of invasion, lymph node metastasis, abdominal cavity metastasis, abdominal metastasis, ascites and adjacent organ invasion of the primary tumor. The use of diagnostic laparoscopic exploration combined with peritoneal lavage fluid evaluation can significantly improve the accuracy of preoperative staging of gastric cancer[12]. In addition, the eighth edition of AJCC staging of gastric cancer and the prevention and treatment of peritoneal metastasis of gastric cancer have pointed out that laparoscopic exploration

is mainly used in the pre-treatment diagnosis and efficacy evaluation after preoperative treatment of advanced gastric cancer (cT 2-4 and any N and M)[13-14]. At the same time, there is a literature reporting that repeated laparoscopic exploration for patients with neoadjuvant treatment can prevent non-curative laparotomy and better choose treatment methods[15]。

Evidence from randomized controlled clinical trials shows that chemotherapy can significantly improve the efficacy of gastric cancer. ACTS-GC randomized 1059 patients with stage II or III gastric cancer who underwent radical surgery for D2 gastric cancer to the post-surgery adjuvant chemotherapy S-1 or only surgery group. The median follow-up period was 2.9 years. The recurrence rate of the disease was 18.6% (S-1 group) and 23.6% (surgery-only group), respectively, and more than 50% of the recurrences were localized. The 3-year recurrence-free survival rate was 72.2% (in the S-1 group) and 59.6% (in the surgery-only group)[6]。

Systemic chemotherapy provides similar cytotoxic drug levels in normal and tumor tissues and, due to significant dose-limiting toxicity, may prevent the drug within tumor tissue. Those remaining free cancer cells (free cancer cell, FCC) are isolated by abdominal adhesion with relatively poor blood supply. This phenomenon explains at least in part the minimal effect of systemic therapy in locally advanced gastric cancer[16]. Therefore, the choice of treatment options for patients with locally advanced or positive cytology, in addition to standard R0 resection, D2 lymph node dissection and adjuvant therapy, it is necessary to develop other more effective treatment measures in order to further reduce the recurrence rate and cancer-related mortality of advanced gastric cancer. The peritoneal FCC is the root cause and the main cause of peritoneal implant metastasis. Remoof FCC was able to significantly improve the 5-year survival of patients with advanced gastric cancer. Because the peritoneal – plasma barrier limits the efficacy of intravenous administration and direct intraperitoneal administration, cytotoxic agents can increase local exposure and reduce systemic toxic responses. The drug enters the systemic circulation by diffusion from or absorption through the peritoneal lymphatic hole[17-18]. On the other hand, drugs also enter the portal blood by covering the liver, spleen, stomach, small and large intestine and mesenteric surfaces[19]. This route provides treatment for potential

liver micrometastases. Due to the peritoneal-blood barrier, the intraperitoneal peritoneal level is 20-1000 times higher than the plasma level[20]. Huang Wan medium[21]In a phase clinical study, 43 patients with stage b~ gastric cancer (14 with peritoneal metastases) were included to evaluate the clinical efficacy and safety of docetaxel peritoneal perfusion chemotherapy plus CF / 5-FU / OXA (specific protocol: DOC 40mg / m^2The intraperitoneal perfusion d1,8, CF200mg / m2 intravenous infusion d1-5,5-FU375mg / m^2Intravenous infusion of d1 － 5, OXA135mg / m^2An intravenous infusion of d1. One cycle for every 3 weeks, for a total of 2-4 cycles). The data showed that the overall effective (CR + PR) rate of docetaxel peritoneal perfusion chemotherapy and systemic chemotherapy was 58.1%, and the 1-year survival rate was 67.4%, and the patients could tolerate the adverse reactions. Docetaxel peritoneal perfusion chemotherapy for advanced gastric cancer has a good effect and the adverse effects are well tolerated.

A phase II clinical study in Japan[22](PHOENIX-GC: UMIN000005930) found that NIPS treatment: S-1 orally in combination with paclitaxel (paclitaxel, PTX) by intravenous (intravenous, IV) and intraperitoneal (intraperitoneal, IP) (1 course every 3 weeks: IV infusion of PTX (50 mg / m^2) And injected PTX (20 mg / m into the peritoneal cavity^{2}Solsolution in 1000 mL normal saline), oral S-1 80 mg / (m2 • d) for 7 d), which can significantly improve the transformation effect of patients with peritoneal metastasis of gastric cancer. Although its phase III multicenter randomized controlled study, the results showed with conventional systemic chemotherapy regimen gigio / cisplatin (intravenous cisplatin, 60 mg / m^2, d 8, oral Tigio, 80 mg / (m^2 • D), d 1~21, once every 5 weeks), this protocol does not show advantages, possibly due to the deviation of peritoneal metastasis and the resulting ascites in the two groups due to the participation of many medical centers. However, subgroup analysis showed that NIPS regimen achieved good efficacy for female patients with peritoneal metastasis gastric cancer with diffuse histology or with obvious ascites. In recent years, several studies in Japan show that for advanced gastric cancer with peritoneal metastasis, the median survival of traditional systemic palliative chemotherapy hovering around 1 year, and receive NIPS treatment with survival up to 17.6 months, even 23.6 months, a year survival rate also reached 80%, conversion

surgery rate can reach more than 50%. In addition, Professor Zhu Zhenggang from Shanghai Ruijin Hospital is carrying out a prospective multi-center randomized controlled (DRAGON-GC) study on NIPS therapy for patients with gastric cancer peritoneal metastasis, so as to predict the efficacy of NIPS, and then realize the individualized comprehensive treatment of gastric cancer patients. At present, the preliminary study results are effective and safe.

.2 1 Karelizumab for gastric cancer

Carlizumab is recombinant human anti-programmed death molecule 1 (programmed death-l, PD-l), PD-1 is a protein receptor expressed on the surface of T cells, involved in the apoptotic process, PD-l belongs to the CD28 family, and cytotoxic T lymphocyte antigen 4 (cytotoxic T lymphocyte antigen 4, CTLA-4) has 23% amino acid identity, but its expression is different from CTLA-4, mainly expressed on activated T cells, B cells and myeloid cells. PD-1 has two ligands, namely PD-L1 and PD-L2. PD-L1 is mainly expressed on T cells, B cells, macrophages and dendritic cells (dendritic cell, DC), and it can be upregulated on the cells after activation. However, the expression of PD-L2 is relatively restricted and is mainly expressed on antigen-presenting cells, such as activated macrophages and dendritic cells. Humanized anti-PD1 monoclonal antibodies are able to bind specifically to PD-1 and block the interaction between PD-1 and its ligand, allowing T cells to restore the immune response against the tumor.

Based on the data from the results of a phase II single arm clinical study of carizumab in relapsed refractory cHL, CFDA approved on 29 May 2019 for marketing in patients with relapsed or refractory classical Hodgkin lymphoma after at least second-line systemic chemotherapy 2. Carilizumab has carried out related trials in several species, and has achieved certain efficacy. In 2018, huang et al published in the journal C ancer cary bead single second-line and above treatment of advanced gastric cancer and esophageal and gastric junction cancer phase I study, the results in 30 patients, 1 case achieved complete remission, objective response rate reached 23.3%, disease control rate of 43.3%, the median progression-free survival is 8 weeks, adverse reactions are mainly grade 1-2, controllable[23]. In 2019, Xu Jianming et al. published in Clin Cancer Res a clinical study of carilizumab and apatinib for gastric cancer or gastroesophageal cancer. As a result, 25 patients were included, the objective response rate reached 17.4%, the disease control rate reached 78.3%, the

median progression-free survival was 2.9 months, and the median survival was 11.4 months[24]. Shen Lin et al. conducted the clinical study of carlizumab and Cape OX sequential carlizumab and apatinib for advanced gastric cancer, and the objective response rate was 58.3%, m D o R 5.7 months, m OS 14.9 months and m PFS 6.8 months[25]。

Due to advanced gastric cancer surgery after peritoneal planting incidence of metastasis rate as high as more than 50%, is one of the main reasons affecting the prognosis, the center early to shed cell positive gastric cancer patients to carry out many clinical studies, respectively for pure SOX chemotherapy, HIPCE and SOX chemotherapy, HIPEC and apatinib mesylate plus giol chemotherapy[26], The R0 resection rates in the three groups were 0%, 14.3%, and 42.11%, respectively, and the one-year overall survival rate in the three groups was 58.3%, 71.4%, and 65.79%. It can be seen that with the intervention of intraperitoneal warm chemotherapy and targeted therapy, the R0 resection rate and 1-year overall survival rate of such patients have gradually improved, but the overall R0 resection rate and survival rate are not ideal. Another study of peritoneal metastasis of gastric cancer included 32 patients[27]In patients with gastric cancer peritoneal metastasis (P 1 CY 1) combined with intraperitoneal topical plus systemic chemotherapy NIPS and apatinib transformation, the objective response rate was 62.50% and DCR was 90.63%. All patients underwent secondary laparoscopic exploration, and the PCI score decreased in 24 (75.00%) and 8 (25.00%), of which the R0 resection rate was 18.75%. The median follow-up time was 25.2 months, mOS was 16.2 months, and mPFS was 14.9 (11.4 to 20.3) months. Common adverse reactions mainly include myelosuppression, peripheral sensory neuropathy, digestive tract reaction and oral mucositis, with no deaths related to adverse reactions. This study combination can improve the R0 resection rate and disease control rate of peritoneal metastatic gastric cancer patients, and the adverse reactions of chemotherapy are controllable, which is a safe and effective conversion treatment. In order to further study the efficacy of immunotherapy for gastric cancer transformation and further improve the survival rate of R0 resection and survival, the NIPS combined with carlizuAb and Tio in patients with exfoliated cell-positive gastric cancer.

2. Study objectives, content, and significance

Primary objective: To evaluate the R0 resection rate and turning rate of NIPS combined with carilizumab and tigio transformation in the treatment of exfoliated cytology positive gastric cancer alone.

Secondary objectives: (1) safety

(2) The 3-year PFS

(3) A 3-year OS

（4）ORR，DCR

(5) The TRG grade

Exploratory objectives: Whether biomarkers are associated with treatment efficacy (including but not limited to PD-L1 expression, T cell subsets, etc.)

Study content: To evaluate the efficacy and safety of NIPS combined with carilizumab and Taggio for conversion therapy in patients with positive shedding cytology.

Study significance: through the prospective clinical study of NIPS combined with carilizumab and tigio for the transformation treatment of shedding cytology positive gastric cancer, further explore the value of NIPS combined with carilizumab and tigio in the treatment of advanced metastatic gastric cancer, and provide clinical basis for the best comprehensive treatment plan.

3. implementation plans

research design

(I) Study subjects:

Only patients with positive intraperitoneal cytology (CY 1P 0).

(2) Study design

The study was a clinical phase exploratory study that included patients with gastric cancer confirmed as CY 1 P 0 after laparoscopic exploration, and all patients received NIPS combined with carlizumab and tigiol, drug selection and dose:

Carrelizumab: 200m g / time, intravenous 30min infusion (not less than 20min and not more than 60min), q 3w. A total of 4 cycles.

NIPS with albumin paclitaxel: intraperitoneal dose of 80mg / m^2, D 1; intravenous dose of 180mg / m^2,d1. Every 21 days is one treatment cycle for 4 cycles.

Tigio: BSA <1.25m^2，40mg bid，1.25m^2≤BSA≤1.5m^2，50mg bid，BSA >1.5m^2,60mg bid, the first dose was taken on the first night, and the last dose

was taken in the morning on day 15 with intermittent chemotherapy.1 cycle every 3 weeks, 2 weeks per gigiintake, 1 week rest for 4 cycles.

In operable patients, the original regimen was continued for adjuvant therapy for 4 cycles, after which carelizumab monotherapy was maintained until 1 year. Inoperable patients are recommended by the investigator according to the guidelines.

(3) Research indicators

Primary study endpoint: R0 resection rate, negative conversion rate.

Secondary study endpoints: 1. Safety

And 2. 3 years of PFS

. 3 3 OS

. 4ORR，DCR

5. The TRG grade No

Exploratory endpoints: relationship between biomarkers and efficacy prediction (including but not limited to PD-L1 expression, T cell subsets, etc.)

(4) Statistical methods

As an exploratory trial, 30 cases are planned to be enrolled.

Mean, standard deviation, median, minimum and maximum values were calculated for age, height, weight, and tumor clinical and pathological data according to their distribution. The number of cases and percentage of various categories were calculated for the classification indexes such as gender and postoperative complications. All statistical analyses were performed using SPSS21.0 and all tests were two-sided with significance level α =0.05.

technology roadmap

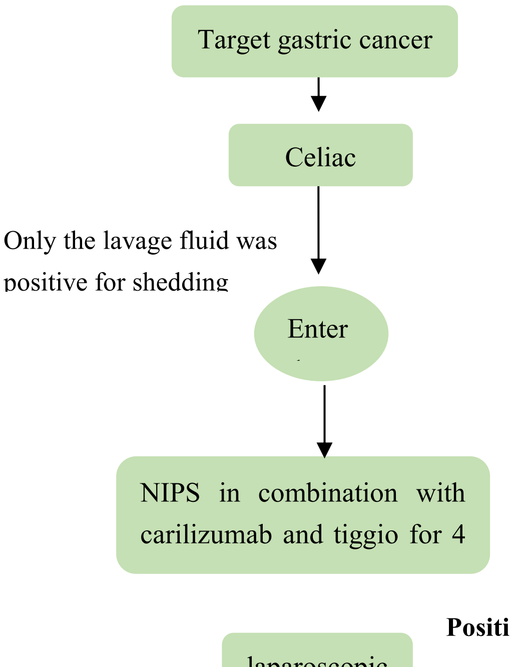

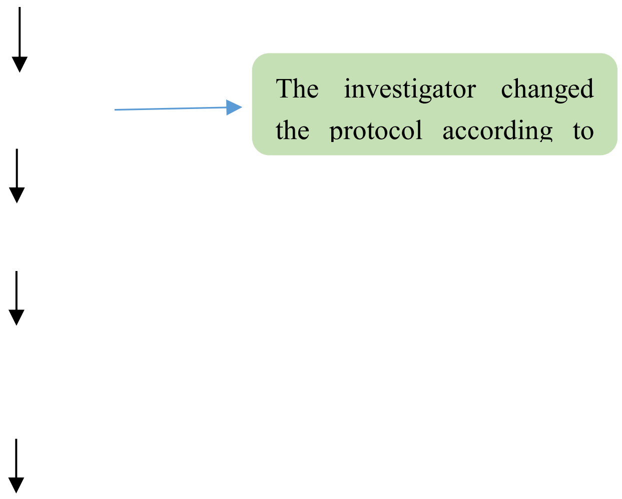

Implementation steps

(I) Inclusion criteria

1. Primary treatment patients, without chemotherapy, radiotherapy or other anti-tumor therapy before the start of the clinical trial;
2. Age: 18-70 years old;
3. Male or non-pregnant or lactating women;
4. Gastroscopy and pathology confirmed gastric adenocarcinoma, and the immunohistochemical test was negative for HER-2;
5. Imaging examination confirmed T stage as T3 and T4, and no macroscopic distant metastasis; positive cytology;
6. The blood routine meets the following conditions: white blood cell count 3.510^{9}/ L, 1.510 for neutrophils^{9}/ L, platelet count of 10010^{9}/ L, hemoglobin of 90 g / L;
7. Biochemical examination meets the following conditions: ALT 2.5 upper limit of normal value (ULN), AST 2.5 ULN, serum total bilirubin 1.5 ULN, blood creatinine 1.5 ULN;
8. LV ejection fraction 50%;
9. ECOG score: 0-1;

10 Be able to comply with the protocol during the study period and voluntarily sign the informed consent form.

(2) Exclusion criteria

1. Use of immunosuppressive drugs within 14 days prior to the use of carlizumab, excluding nasal and inhaled corticosteroids or physiological doses of systemic steroid hormones (i. e., no more than 10 mg / day of prednisolone or other corticosteroids at equivalent physiological doses);

2. With carlizumab, there was any active autoimmune disease or autoimmune history (including but not limited to autoimmune hepatitis, pneumonia, interstitial pneumonia, uveitis, enteritis, hepatitis, pituitary, vasculitis, nephritis, hyperthyroidism, reduced thyroid function; vitiligo or asthma in childhood, adults without any intervention; asthma requiring medical intervention with bronchodilators could not be included);
3. History of other malignancies within 5 years;
. 4 patients with human immunodeficiency virus (HIV) infection or known acquired immunodeficiency syndrome (AIDS), active hepatitis B (HBV DNA 1000 IU / ml), hepatitis C (hepatitis C antibody positive and HCV-RNA above the lower limit of detection of the analytical method) or combined hepatitis B and hepatitis C coinfection requiring antiviral treatment during the study;
6. Other transferred organs;
7. Severe or uncontrolled medical diseases and infected persons (including atrial fibrillation, angina pectoris, cardiac insufficiency, ejection fraction less than 50%, difficult to control hypertension, etc.);
8. Patients with a history of psychiatric substance abuse and unable to quit or with mental disorders;
9. Severe or uncontrolled mental illness;
10. Patients with concomitant diseases that seriously endanger patient safety or affect the patient to complete the study according to the discretion of the investigator;
11.

(3) Exit criteria

1. Allergic reaction occurs

2. patient requested withdrawal

3. Other situations requiring the termination of the study

(4) Elimination criteria

1 Patients who failed to complete the trial plan

2. Patients who violate the requirements of the trial protocol

3. Patients with poor data record quality, incomplete data and inaccurate data

(V) Screening and enrollment stage

1. Screening of subjects who meet the criteria

2. Fully inform the patient or authorized person and obtain the informed consent form signed by the selected subject or their guardian / legal representative.

3. Coeliac exploration:

①②③Coeliac exploration (open abdominal exploration or laparoscopic exploration). The exploration included the following methods: abdominal viscera and peritoneal palpation, laparoscopic macroscopic examination, and detection of peritoneal free cancer cells. Full washing, agitation and collection with 800 mL of saline. During flushing, the patient should be in a high and low position, avoid 800 mL of saline from the primary focus, inject from the right upper abdomen, and draw at least 300 mL of flushing fluid from the pelvic cavity. The peritoneal washes was collected and 1 mL of heparin was added to anticoagulation and centrifuged at 1000 g for 10 min. Nucleated cell layers were collected for smear and tumor cells were detected by HE staining.

Abperitoneal shedding cytology findings

Reading by two doctors of pathology department, the positive results of abdominal detached cells of gastric cancer mainly included: large and deep nuclear staining, imbalance of nuclear plasma proportion; dense, rough, uneven distribution, disordered arrangement; nuclear membrane thickening, interruption or wrinkle; increased nucleoli, etc. Positive peritoneal shedding cytology was defined as CY 1.

Enrollment with positive peritoneal shedding cytology.

(VI) Enrollment and post-treatment plan

Patients with gastric cancer confirmed as CY 1 P 0 after laparoscopic exploration were included, and all patients received NIPS combined with carilizumab and tigiol, drug selection and dose:

Carrelizumab: 200m g / time, intravenous 30min infusion (not less than 20min and not more than 60min), q 3w. A total of 4 cycles.

NIPS with albumin paclitaxel: intraperitoneal dose of 80mg / m^2, D 1; intravenous dose of 180mg / m^2,d1. Every 21 days is one treatment cycle for 4 cycles.

Tigio: BSA <1.25m^2， 40mg bid， 1.25m^2≤BSA≤1.5m^2， 50mg bid， BSA >1.5m^2,60mg bid, the first dose was taken on the first night, and the last dose

was taken in the morning on day 15 with intermittent chemotherapy.1 cycle every 3 weeks, 2 weeks per gigiintake, 1 week rest for 4 cycles.

In operable patients, the original regimen was continued for adjuvant therapy for 4 cycles, after which carelizumab monotherapy was maintained until 1 year.

Inoperable patients are recommended by the investigator according to the guidelines.

Monitoring: Related laboratory tests before and after treatment, including routine blood tests, liver and kidney function, tumor markers, myocardial enzymes, troponin, thyroid function, etc.

The toxic side reactions should be observed and evaluated in each cycle.

Every 2 cycles, as judged by the recent efficacy criteria for solid tumors, they were divided into complete response (CR), partial response (PR), stability (SD), and progression (PD). Treatment total response rate was calculated by CR + PR, CR + PR + SD calculates the disease control rate.

2. Surgical treatment

After intraperitoneal treatment with systemic albubupaclitaxel and carilizumab and Tageol, intraperitoneal exploration was performed again, with negative shedding cytology and no macroscopic distant metastasis, and D2 resection was performed.

Distal gastrectomy: showing ligation point of left gastric omenial artery, common hepatic artery, intrahepatic native artery, left gastric artery, ligation point of left and right gastric veins, proximal splenic artery, right lower pyloric arteriovein, and the proximal small curved side.

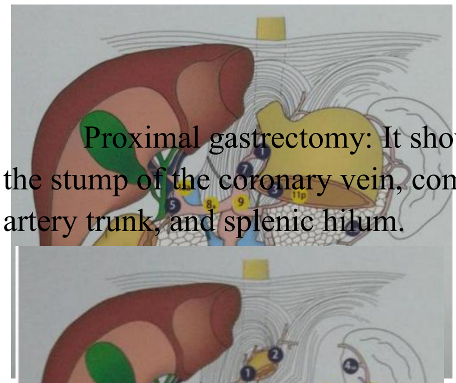

Proximal gastrectomy: It showed the ligation point of the left gastric artery and the stump of the coronary vein, common hepatic artery, proximal splenic artery, celiac artery trunk, and splenic hilum.

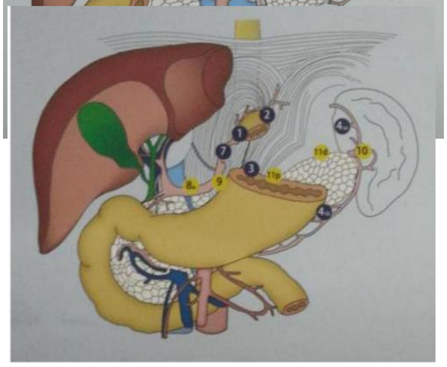

Total gastrectomy: showing the point of left gastric motor and vein ligation, the point of right gastric motor and vein ligation, common hepatic artery, celiac trunk, splenic portal, splenic artery, intrahepatic native artery, right motor and vein ligation of gastric omentum, and lower pyloric arteriovein.

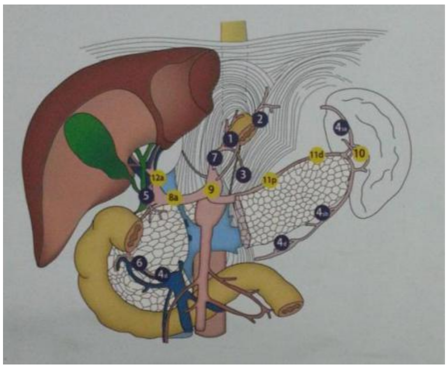

3. After the operation, the D2 lymph nodes should be photographed after dissection.

4. Postoperative adjuvant therapy.

(Vii) Follow-up plan and content

1. Pre-test evaluation

Each subject should complete the following items one week after admission:

(1) Complete medical history data

(2) Detailed personal data

(3) Current treatment situation

(4) Physical examination

(5) Routine blood examination

(6) Liver and kidney function tests

(7) Examination of tumor markers

(8) Cardiac enzymes, troponin, and thyroid function

(9) ECG, gastroscopy, tumor lesion imaging (CT or endoscopic ultrasound)

2. Adverse events:

Adverse events (Adverse Event, AE): any signs, symptoms, syndrome, or deterioration between the subject between the subject of the study and the last visit. Including clinically relevant conditions found in the laboratory or other diagnostic procedures. Adverse events may be: new disease; worsening of symptoms or signs of

treatment status, or worsening of concomitant disease; unrelated to participation in the trial; one or a combination of factors. The term "adverse event" does not imply a causal relationship with the trial.

The main chemotherapy drugs used in this study are albumin, paclitaxel and giio. The main adverse reactions of these chemotherapy drugs are: anemia, leukopenia, granulocytopenia, thrombocytopenia and proteinuria;

Non-hematologic adverse reactions are mainly: nausea, vomiting, diarrhea, peripheral neuritis, sometimes spasm and sensory disturbance around the oral cavity, upper respiratory tract and upper digestive tract. In the intraperitoneal chemotherapy port, there are risks of catheter blockage, drug extravasation, and local infection.

The adverse reactions of carlizumab used in this study are mainly reactive skin capillary hyperplasia.

. 3 Related adverse events of various forms of primary deterioration according to CTCAE v4.03, including:

(1) Adverse events caused by the deterioration of the primary lesion and the peritoneal disseminated lesion:

Digestive tract: loss of appetite, constipation, dehydration, abdominal fullness, heartburn, nausea, digestive tract occlusion, gastrointestinal perforation, digestive tract stenosis, vomiting, low Na aemia, gastrointestinal bleeding.

(2) Adverse events caused by the deterioration of liver metastasis:

Abnormal metabolic / clinical examination values: AST, ALT, bilirubin, alkaline phosphatase.

(3) Adverse events caused by the deterioration of pulmonary metastasis:

Lung / upper respiratory tract: atelectasis, dyspnea, and hypoxemia.

(4) Adverse events caused by the deterioration of other metastatic lesions:

Pain: pain- [metastasis site], hypercalcemia.

(5) Adverse events caused by the deterioration of the systemic status:

Systemic status: fatigue, weight loss, cachexia.

Blood / bone marrow: white blood cells, neutrophils, hemoglobin, platelets.

Cardiovascular system: hypotension.

Edema: head and neck, limbs, trunk / genitalia, viscera.

Metabolic / clinical test values: hypoproteinemia, AST, ALT, acidosis, creatinine, hyperglycemia, hypoglycemia, hyponatremia, hyponatraemia, hyperkalemia, hypokalemia, and other electrolyte disorders.

Lung / upper respiratory tract: pleural fluid (non-malignant), dyspnea, hypoxemia, pulmonary infection.

Renal / urogenital: cystitis, renal failure, oliguria / anuria.

4. Evaluation of adverse events

Evaluation of adverse events / adverse reactions

Comprehensive reference to the CTCAE v4.03 standard. Adverse events were classified by the definition closest to Grade 0-4. For treatment-related death, adverse events of causes of death were classified as Grade 5 in the CTCAE. All adverse events within 30 days after surgery should record the adverse event items, grade and discovery date, and should also be recorded in the case.

For any adverse events arising in the clinical study, medical staff should take necessary measures to ensure the safety and rights of patients, and Grade 4 and above should be immediately reported to the sponsor study unit (except hematological toxicity). The investigator must fill in the serious adverse event form to record the time, severity, duration, measures taken and outcome of the serious adverse reactions.

5. Evaluation after the completion of the treatment

After completion of D2 surgery plus adjuvant chemotherapy, the follow-up was conducted by a dedicated follow-up team, and the first follow-up review began 3 months after surgery. Once every three months for two years, once in the second half of two years, and once a year after five years, long-term follow-up review. Each follow-up review shall include:

(1) routine blood tests and liver and kidney function tests; (2) tumor markers; and (3) tumor status assessment

Blood routine, liver and kidney function indicators and chest X-ray should be performed for each follow-up review, and CT examination is added when the patient has physical abnormalities or tumor markers are elevated. CT examination is recommended for six months within 2 years and one year after 2 years. The first gastroscopy was performed three months (i. e. first follow-up) and annually thereafter.

Tumor markers are recommended to be examined every 3 months for 2 years, one half year after 2 years and one year after 5 years. To evaluate the efficacy, postoperative recovery and near-term side effects.

The evaluation of the effectiveness includes the following aspects:

(1) Tumor efficacy indicators

This includes tumor recurrence (site and time), metastasis (site and time), and death (cause and time).

(2) Evaluation of the long-term survival benefit of the patients

Progression-free survival (Progression Free Survival, PFS): time between randomization and progression (in any respect) or death (for any cause). The appearance of new lesions was used as a criterion for progression, and the marker time point of progression was the date when first detectable new lesions were observed.

Overall survival (Overall survival, OS): This is the time from randomization to death from any cause, which is often considered the best efficacy endpoint in oncology clinical trials. Through follow-up, accurately recorded.

(8) Treatment of tumor recurrence and metastasis

Patients with recurrence were treated according to the current standard clinical pathway.

(IX) Ethical requirements and the informed consent form from the subjects

Written consent from the ethics committee was obtained before the trial starts. Studies must comply with the Declaration of Helsinki and Chinese regulations and regulations regarding clinical studies. Before the subject is enrolled, the investigator will fully introduce to the subject or the legal representative of the subject. And inform the right to withdraw from the trial at any time and under any circumstances. Selected subjects must sign an informed consent form. The investigator shall properly keep all documents approved by the Ethics Committee, including patient informed consent and recruitment materials for supervision.

(10) Progress and completion time of clinical trials

Case collection, treatment were completed within 1 year, and case follow-up was completed within 3 years.

(11) The preservation of the data

All study data and original records during the clinical study shall be kept intact. The study unit should keep this data until 5 years after the end of the study. The data available in this study were kept by the Fourth Hospital of Hebei Medical University.

reference documentation

1.Organization WH: Cancer, Fact sheet N°297, World Health Organization Media centre, Published 2009. Reviewed January 2013.

2.Jemal A, Siegel R, Xu J, et al: Cancer statistics, 2010. CA Cancer J Clin 60:277-300,2010

3.Chen, W., et al., Cancer statistics in China, 2015. CA Cancer J Clin, 2016.

4.Cunningham D, Allum WH, Stenning SP, et al: Perioperative chemotherapy versus surgery alone for resectable gastroesophageal cancer.N Engl J Med 355:11-20, 2006

5.Bang YJ, Kim YW, Yang HK, et al: Adjuvant capecitabine and oxaliplatin for gastric cancer after D2 gastrectomy (CLASSIC): a phase 3 open-label, randomised controlled trial.Lancet 379:315-21, 2012

6.Sakuramoto S, Sasako M, Yamaguchi T, et al: Adjuvant chemotherapy for gastric cancer with S-1, an oral fluoropyrimidine.N Engl J Med 357:1810-20, 2007.

7.Sasako M, Sano T, Yamamoto S, et al: D2 lymphadenectomy alone or with para-aortic nodal dissection for gastric cancer.N Engl J Med 359:453-62, 2008.

8.D'Angelica M, Gonen M, Brennan MF, et al: Patterns of initial recurrence in completely resected gastric adenocarcinoma.Ann Surg 240:808-16, 2004.

9.Ribeiro U, Jr., Gama-Rodrigues JJ, Safatle-Ribeiro AV, et al: Prognostic significance of intraperitoneal free cancer cells obtained by laparoscopic peritoneal lavage in patients with gastric cancer.J Gastrointest Surg 2:244-9, 1998.

10.Mori N, Oka M, Hazama S, et al: Detection of telomerase activity in peritoneal lavage fluid from patients with gastric cancer using immunomagnetic beads.Br J Cancer 83:1026-32, 2000.

11.Ikeguchi M, Oka A, Tsujitani S, et al: Relationship between area of serosal invasion and intraperitoneal free cancer cells in patients with gastric cancer.Anticancer Res 14:2131-4, 1994.

Zhu Xinqiang, Li Ziyu. The value of laparoscopic exploration combined with lavage biopsy in the preoperative staging of advanced gastric cancer [J]. World Chinese Digestion Magazine, 2012 (25): 2407-2409.

13. Ji Jiafu, Shen Lin, Xu Huian, et al. Chinese expert consensus on the prevention and treatment of peritoneal metastasis in gastric cancer [J]. Chinese General Surgery Literature (electronic version), 2017,9 (5): 29-40.
14.Doescher J, Veit J A, Hoffmann T K.The 8th edition of the AJCC Cancer Staging Manual[J].HNO, 2017:1-5.
15.Cardona K, Zhou Q, Gönen M, et al.Role of repeat staging laparoscopy in locoregionally advanced gastric or gastroesophageal cancer after neoadjuvant therapy[J].Annals of Surgical Oncology, 2013, 20(2):548-554.
16.Stewart JHt, Shen P, Levine EA: Intraperitoneal hyperthermic chemotherapy for peritoneal surface malignancy: current status and future directions.Ann Surg Oncol 12:765-77, 2005.
17.Bettendorf U: Lymph flow mechanism of the subperitoneal diaphragmatic lymphatics.Lymphology 11:111-6, 1978.
18.Bettendorf U: Electronmicroscopic studies on the peritoneal resorption of intraperitoneally injected latex particles via the diaphragmatic lymphatics.Lymphology 12:66-70, 1979.
19.Katz MH, Barone RM: The rationale of perioperative intraperitoneal chemotherapy in the treatment of peritoneal surface malignancies.Surg Oncol Clin N Am 12:673-88, 2003.
20.Van der Speeten K, Stuart OA, Sugarbaker PH: Pharmacology of perioperative intraperitoneal and intravenous chemotherapy in patients with peritoneal surface malignancy.Surg Oncol Clin N Am 21:577-97, 2012.
21. Huang Wanzhong, Jiang Hua, Liu Yanwen, et al. Clinical study of docetaxel peritoneal perfusion chemotherapy for advanced gastric cancer [J]. Modern Medical Oncology, 2011,19 (5) 960-963
22.Kitayama J, Ishigami H, Yamaguchi H, et al.Salvage gastrectomy after intravenous and intraperitoneal paclitaxel (PTX) administration with oral S-1 for peritoneal dissemination of advanced gastric cancer with malignant ascites[J].Annals of Surgical Oncology, 2014, 21(2):539-546.
23. Huang J, Mo H, Zhang W, et al.Promising efficacy of SHR-1210, a novel anti-programmed cell death 1 antibody, in patients with advanced gastric and gastroesophageal junction cancer in China[J].Cancer.2019, 125(5): 742-749.
24. Xu J M, Zhang Y, Jia R, et al.Anti-PD-1 Antibody SHR-1210 combined with Apatinib for Advanced Hepatocellular Carcinoma, Gastric or Esophagogastric

Junction Cancer: An Open-label, Dose Escalation and Expansion Study[J].Clin Cancer Res.2019, 25(2): 515-523.
25. Peng Z, Wei J, Wang F, et al.Camrelizumab combined with chemotherapy followed by camrelizumab plus apatinib as first-line therapy for advanced gastric or gastroesophageal junction adenocarcinoma[J].Clinical cancer research : an official journal of the American Association for Cancer Research:clincanres.4691.2020.
26. Ding Pingan, Yang Peigang, Pastoral, etc. Efficacy of peritoneal heat infusion of paclitaxel combined with apatinib and tigiol chemotherapy in patients with positive gastric cancer with shed cytology [J]. Journal of Practical Medicine, 2021,37 (4): 7.
27. Ding Ping An, Yang Peigang, Pastoral, etc. Efficacy of peritoneal heat perfusion chemotherapy combined with systemic chemotherapy and apatinib transformation therapy on peritoneal metastasis of gastric cancer [J]. Chinese Oncology Clinical, 48 (8): 6.

Appendix 1 Standardized operation procedures for radical gastrectomy

In the implementation of radical gastrectomy, the standardized operation process of radical gastrectomy must be clarified, so as to determine that radical gastrectomy is the most suitable surgical method for patients and ensure the success of radical gastrectomy.

D2 radical resection is the standard procedure for gastric cancer, with tumor invasion depth beyond the submucosa (muscle layer or above),

Or with lymph node metastasis but has not yet invaded the adjacent organs, should undergo standard surgery (D2 radical surgery, 10 groups of lymph node dissection needs to be marked).

Table 1. Range of lymph node dissection in D1 and D2 (standard radical resection) in different sites

	A distal gastrectomy was performed	The proximal gastrectomy	total gastrectomy
D1	1、3、4sb 、4d 、5、6、7	1、2、3、4sa 、4sb 、7	1-7
D2	D1+8a 、9、11p 、12a	D1+8a 、9、10、11	D1+8a 、9、10、11、12a

Standardized operation procedure of radical gastrectomy:

1. Incision and incision protection: the middle of the upper abdomen to facilitate exposure.
2. Access, exploration: according to the principle from far to far, focus on the liver, peritoneal, pelvic, upper mesenteric vascular roots and periabdominal aortic lymph nodes. If there was significant metastasis or enlarged lymph nodes at the above sites, D2 lymph node dissection was abandoned for palliative resection.
3. Cut off the ligament in the left outer lobe of the liver: if a total gastrectomy, first cut the ligament in the left outer lobe of the liver (trigonal ligament, coronary ligament) in order to expose the right side of the cardia and the small omentum.
4. Free omentum and anterior lobe of transverse mesentery: in order not to damage the spleen during the operation, put a wet gauze pad behind the spleen in front of the free omentum, and pad the spleen, so that it is moved to the middle of the abdominal cavity and lifted to the upper part.

5. Ligation of the left blood vessel of the gastric omentum to clean the lymph nodes of the left group of the large gastric curvature: with the upward separation of the anterior lobe to the lower edge of the pancreas, the left artery of the gastric omentum was severed, and the lymph nodes of the left group of the large gastric curvature were cleaned.

6. Separate the gastric colonic vein to remove the mesenteric para venous lymph nodes and cut the right gastric omentum: remove the colon in the colon and the gastric colon, find the middle colon vein and superior mesenteric vein, to remove the lymphoid adipose tissue around the superior mesenteric vein. Along the gastric colon, the beginning of the right vein of the gastric omentum was found, and the right vein of the gastric omentum was ligated and severed at the root.

7. In the upper edge of the pancreas right gastroduodenal artery, cut off the right artery, sweeping the pyloric lymph nodes: transverse mesentery anterior lobe in the lower edge of the pancreas and the pancreatic capsule, further from the lower edge of the pancreas to the upper edge of the pancreas, the middle pancreas to the duodenal free pancreatic capsule, until the gastroduodenal artery, find the artery down to find the right artery (the artery is the end of the gastroduodenal artery branch), in the root ligation, cut off the gastric mesh right artery, cleaning the pyloric lymph nodes.

8. Cleaning of the hepatic duodenal ligament along the hepatic artery lymph nodes: after the right gastroomental artery was cut, the stomach and the omentum were further pulled upward, while the pancreas was gently pulled downward and backward, tracking up the gastroduodenal artery to find the common hepatic artery and the native hepatic artery.

9. Cut off the duodenum: free ligation, cut off the small blood vessels and adipose tissue between the pancreas and the duodenum, fully free from the duodenum. Cut and close the duodenum with a linear cutter.

10. Total hepatic artery lymph node dissection: the stomach to the left, with wet gauze under the pancreas, between the upper edge of the pancreas and the common hepatic artery, since the liver artery to the left, because the common hepatic artery and the upper edge of the pancreas between a small blood vessels, and damage the pancreas is prone to pancreatic leakage, the cleaning to careful ligation.

11. Clean the abdominal and left gastric artery peripheral lymph nodes: the cleaning free lymphoid fat tissue and the stomach further to the left pull, the abdominal aorta right after the peritoneal incision to the midline, show the right

diaphragm angle, remove the abdominal right front nerve lymphatic fat tissue, at the same time remove the left gastric artery lymphatic fatty tissue, in the root ligation, cut off the left gastric artery. There is a solid nerve plexus between the celiac trunk and the superior mesenteric artery, so the nerve plexus cannot be removed clean, otherwise diarrhea, abdominal distension and the quality of life will decline after surgery.

12. Cleaning the lymph nodes around the splenic artery: because the splenic artery is distorted and partially wrapped in the pancreatic tissue, and the splenic vein is walking under the splenic artery, the splenic vein should not damage the splenic vein and pancreatic tissue during dissection. Further turn the stomach to the upper left, press the pancreas downward with gauze, and lift the proximal splenic artery with a vascular traction belt to clean the lymphatic adipose tissue, and the posterior gastric artery should be ligated and cut off. Generally, cleaning the proximal splenic artery is not required to clean the splenic artery. In the process of cleaning the native hepatic artery, the common hepatic artery, and the splenic artery, the arterial blood sheath should be opened.

13. Clean the lymph nodes on the right and small side of the cardia: pull the stomach down to the right, and clean down the esophagus of the diaphragm with a right-angle forceps. First, clean the lymphatic adipose tissue of the anterior wall of the small wall of the stomach, and down to the predetermined gastric resection line. In the same way, remove the lymphatic adipose tissue of the posterior wall of the small side of the stomach to the scheduled gastric resection line. The vagal trunk was cut during dissection around the cardiac esophagus.

14. The scope of gastric resection was determined according to the tumor site and size, and the corresponding digestive tract reconstruction was performed simultaneously.

Adp. 2 Dose adjustment for toxicity

2. 1 Routine precautions for dose adjustment

Expected toxicities and subscales were described in the treatment regimen. Toxicity will be graded according to the National Cancer Institute (NCI) Common Toxicity Criteria (CTC) version 4.03.

Treatment delay or dose adjustment (if after adverse reactions) is as follows. The reasons for dose adjustment or delay, supportive care measures taken, and their results will be recorded on the patient record card and the CRF.

General Notes:

For adverse effects at baseline, the dose may be adjusted according to the change of toxicity if deemed necessary by the investigator. For example, if a patient has a baseline frailty level 1 and develops to level 2 during treatment, the dose should be adjusted for level 1 toxicity.

For toxicity (such as alopecia, appetite changes, etc.) considered by the investigator as unlikely to develop a severe or critical event, treatment will be continued at the original dose without reduction or interruption. In addition, anemia (non-hemolytic) does not require reduce or interrupt treatment, as it can be improved with transfusion.

When several different levels or severity occur simultaneously.

If delayed or interrupted for more than three weeks, the patient will be withdrawn from the study due to toxicity.

If a patient has a non-haematological adverse event of severity grade 3 or greater than grade 3, if it is not possible to continue the original dose or reduced treatment, discuss with the Sponsor before continuing treatment.

2.2 Dose adjustment of Tiggio

2.2.1 Dose adjustment for haematological toxicity

The dose of Tiggio was adjusted simultaneously according to the guidelines in Tables 1,2 and 3.

It is generally believed that Tiggio does not aggravate neutropenia / granulocyte deficiency or excessively prolong its duration. Therefore, if a hematologic toxicity of <grade 2 is found in an unplanned laboratory examination during the chemotherapy cycle, the io will continue. In addition, if grade 3 or haematological 4 toxicity occurs during the chemotherapy cycle. The next cycle of chemotherapy can only start when

the haematological toxicity returns to <grade 1. For anemia (non-hemolytic), there is no dose reduction or treatment interruption because it can be treated satisfactorily with a blood transfusion.

Table 1 Dose adjustment for febrile neutropenia during Tiggio chemotherapy

	**Level 3**<br>***ANC < 1.0x10^9/L companion***<br>***≥Body temperature was 38.5°C***	**Level 4**<br>***ANC < 1.0x10^9/ L, with a fever of 38.5°C≥***<br>***And a life-threatening sepsis***
The first occurrence	75% of the original dose of Tigio	Stop treatment permanently unless 50% of the original dose of Tigio is the best treatment option for the patient.
The second occurrence	Stop treatment permanently unless 50% of the original dose of Tigio is the best treatment option for the patient.	Treatment was permanently discontinued
The toxicity (except anemia) must be mitigated to grade 1 (i. e. ANC> 1.5 x10^9/ L, platelets> 75x10^9/ L) to start chemotherapy.		

Table 2 Dose adjustments for neutropenia during Tiggio chemotherapy

	**Level 2**<br>***1.0<ANC<1.5 x10^9/L***	**Level 3**<br>***0.5<ANC<1.0 x10^9/L***	**Level 4**<br>***ANC< 0.5 x10^9/L***
**Laboratory values before the start of the chemotherapy cycle: deferred chemotherapy until ANC 1.5 x 10**≥^{9}/ L, platelets 75 x10≥^{9}/ L and non-hematologic toxicity returned to baseline or grade 1 and then chemotherapy at the dose specified below			
The first occurrence	Doses need not be adjusted	75% of the original dose of Tigio	50% of the original dose of Tigio
The second occurrence	Doses need not be adjusted	75% of the original dose of Tigio	Treatment was permanently discontinued
The third occurrence	Doses need not be adjusted	Permanently for cessation of treatment unless it is in the best interest of the patient that the investigator considers that single-agent chemotherapy with the original dose of 75% is the best treatment option for the patient.	not applicable

Table 3 Dose adjustment for thrombocytopenia and anemia during Tiggio chemotherapy

**thrombocytopenia**	**≥Platets 50- <75 x10^9/L**	**blood cells ≥25 - < 50 x10^9/L**	**blood cells < 25 x10^9/L**
**Laboratory values before the start of the chemotherapy cycle: deferred chemotherapy until ANC 1.5 x 10**≥^{9}/ L, platelets 75 x10≥^{9}/ L, non-hematologic toxicity returns to baseline or level 1 and then start chemotherapy at the following doses			
The first occurrence	The dosage of Tgiol was unchanged	Tiggio was reduced by 1 dose level	Tiggio was decreased by 2 dose levels
The second occurrence	The dosage of Tgiol was unchanged	Tiggio was reduced by 1 dose level	Stop treatment permanently unless the investigator considers that chemotherapy should be continued in the best interest of the patient<br>(Tio reduces 2 dose levels)
The third occurrence	The dosage of Tgiol was unchanged	Tiggio was decreased by 2 dose levels	Treatment was permanently discontinued
**Anemia (non-hemolytic) at any time during treatment**	**Grade 2 hemoglobin 8.0 - < 10.0 g/dL**	**Grade 3 hemoglobin 6.5 - < 8.0 g/dL**	**Grade 4 hemoglobin was <6.5 g / dL**
Any time it happens	No dose adjustment (corrected by transfusion)	No dose adjustment (corrected by transfusion)	No dose adjustment (corrected by transfusion)

2.2.2 Dose adjustment for Tigorol hematologic toxicity

If a Grade 2,3 or 4 non-haematological toxicity occurred, Tiggio was then stopped immediately continued (unless this toxicity was related only to oxaliplatin) and further action was taken according to the instructions below (see Table 4).

This section describes the Tiggio dose adjustment rule in the event of an associated toxicity. The tigio dose should not be reduced in case of sensory neurotoxicity.

Chemotherapy should be stopped if the creatinine clearance drops to <30 mL/min during chemotherapy.

Specific reduction methods (i. e. number of tablets taken by patients at different dose levels) are shown in Annex 4. Once the amount is reduced, no more amount should be added later, unless oxaliplatin is permanently discontinued.

Note: The suspension of chemotherapy is the loss of treatment days, and chemotherapy should be continued according to the scheduled chemotherapy schedule. Doses missed by interrupted chemotherapy will not be replaced.

Table 4 Tigio dose adjustment for non-hematologic adverse events

Note: Grade 2 toxicity must stop chemotherapy and must not be continued until the toxicity returns to grade 1

	**Level 2**	**Level 3**	**Level 4**
**The first occurrence**	No reduction;<br>Prevention if possible	(Tiggio: 1 dose level reduction), prophylaxis if possible	Permanent discontinued unless the investigator considers for the best interest of the patient to continue chemotherapy (gigio: 2 dose level reduction)
**The second occurrence**	(Tiggio: 1 dose level reduction)	(Tiggio: reduced by 2 dose levels)	
**The third occurrence**	(Tiggio: reduced by 2 dose levels)	Permanent discontinuation of treatment unless chemotherapy is continued by the investigator for the patient's best interest	
**The fourth occurrence**	Discontinue chemotherapy permanently unless the investigator thinks it should be continued in the best interest of the patient.		

2.2.3 Grade 2 diarrhea

Tiggio causes diarrhea, sometimes severe. Patients with severe diarrhea should be closely monitored, and if dehydration occurs, water and electrolyte should be replenished in time. If Grade 2,3, or 4 diarrhea occurs, the Tiger treatment should be interrupted immediately until the diarrhea resolves or decreases to grade 1. With the second grade 2 or more toxicity, the subsequent dose of Tiggio should be reduced. Appropriate anti-diarrhea standard treatment (e. g., loperamide) should be started

early after the onset of diarrhea. Must wait for diarrhea to ease grade 0 or 1 and stop loperamide for 24 hours before starting Tgio chemotherapy.

2.2.4 Grade 2 nausea / vomiting

Tiggio may cause nausea or vomiting. If grade 2,3 or 4 nausea and / or vomiting occurs, gigio chemotherapy should be interrupted immediately until these symptoms resolve or grade 1. Symptomatic treatment is required for nausea / vomiting. To prevent nausea / vomiting, patients should be given oral antiemetic medication allowing for self-administration during nausea and vomiting at home. Oral metoclopramide (metoclopramide) may also consider a 5-HT 3 antagonist. In the event of nausea or vomiting, adequate secondary treatment and prophylactic treatment should be given. If adequate precautions or nausea / vomiting occurs, the dose should be adjusted per Table 4.

2.2.5 Grade 2 hand-foot syndrome

Hand and foot syndrome (paw foot swelling and pain or chemotherapy-related limb erythema) is a skin toxicity with a severity score of grades 1-3, as described below:

Grade 1: painless skin changes or dermatitis (e. g. erythema, desquamation).

Grade 2: skin changes with pain, without dysfunction.

Grade 3: skin changes with pain, with dysfunction.

If grade 2 or 3 hand-foot syndrome occurs, tigio chemotherapy should be interrupted immediately until the toxicity resolves or decreases to grade 1.

Hand and foot syndrome should be treated symptomatic (emulsion application is recommended). Treatment with vitamin B6 or secondary prevention of hand and foot syndrome was not permitted, as concurrent use of vitamin B6 and cisplatin was previously reported.

2.2.6 Grade 2 stomatitis

If grade 2 or 3 stomatitis occurs, tigio chemotherapy should be interrupted immediately until the toxicity resolves or decreases to grade 1.symptomatic treatment.

2.2.7 Cardiotoxicity

In case of grade 2 cardiotoxicity, the patient should permanently disthe giio chemotherapy.

2.3 Delay of chemotherapy

The chemotherapy should be postponed until:

≥ Neutrophil count was 1,500 / mm^3≥And a platelet count of 75,000 / mm^3。

Any non-hematologic toxicity (except alopecia) returned to baseline or grade 1.≤

Toxicity: If toxicity delays one or all test drugs for more than three weeks, the patient should be withdrawn from the study.

2.4 Albumin Paclitaxel Dose Adjustment

veterinary drug	dose level	dosages
**Albumin Paclitaxel (IV)**	Level 0 (full volume)	180mg/m^2
	Level-1	150mg/m^2
	Level-2	100mg/m^2
**Albumin paclitaxel (intraperitoneal)**	Level 0 (full volume)	80mg/m^2
	Level-1	50mg/m^2

2.5 The dose adjustment of Carelizumab

The adverse events caused by immune tumor (Immuno-Oncology, I-O) drugs differ from other types of anti-tumor drugs, severity and duration and particularity. SHR-1210 belongs to this class of drugs, so it is necessary to identify and treat its caused adverse events early to reduce the occurrence of serious toxic events. Referring to the safety rules of similar products in China to assist the investigator in evaluating and handling the adverse events of the following systems: gastrointestinal tract, kidney, lung, liver, endocrine, skin and nerve. Rules for the safety of immune tumor drugs refer to the C SCO immune checkpoint inhibitors.

Since carilizumab is a fully humanized monoclonal antibody with less possibility of infusion or allergic reaction, a preventive medication is generally not needed before the infusion of carilizumab. Based on the published information, the most likely allergic reaction / allergy event occurred within 24 hours of the infusion. If it occurs, the infusion should be slowed or interrupted, clinical supportive therapy,

and preventive medication should be given before later medication. Possible allergic reactions include fever, chills, chills, headache, skin rash, joint pain, abnormal blood pressure, or bronchospasm.

For management of anaphylaxis, according to institutional medical practice and guidelines. The following are treatment recommendations for infusion reactions for reference.

CTCAE rank	clinlcal symptom	Recommended processing	Treatment with SHR-1210
Level 1	Mild transient response	Bedside was observed and closely monitored until recovery. Pre-infusion prophylaxis is recommended: 50 mg, or equivalent and / or acetaminophen 325-1000 mg at least 30 min before SHR-1210.	Continue to use
Level 2	Moderate response, requiring treatment or suspension, and can be quickly relieved after symptomatic treatment (such as antihistamines, non-steroidal anti-inflammatory drugs, anesthetics, bronchodilator	Normal saline intravenous infusion, diphenhydramine 50 mg IV or equivalent and / or acetaminophen 325-1000 mg; observed at the bedside and monitored closely until recovery. Corticosteroids or bronchodilators may be considered as clinically indicated; original medical records documented the amount of study drug infusion; later recommended post-infusion prophylaxis: 50 mg or equivalent and / or acetaminophen 325-1000 mg at least 30 min before SHR-1210. If necessary, cortisolhormone (hydrocortisone dose equivalent to 25 mg) may be used.	suspend. When re-medication after symptom resolved, an initial infusion speed of 50% was used. If there are no complications within 30 minutes, it can return to the original infusion rate. Watch closely. If symptoms recur, no infusion was given

	s, intravenous fluids		
Adventitia level 3	Grade 3: severe response, no rapid response after treatment and / or suspension; or recurrence of symptoms after remission; sequelae requiring hospitalization. Level 4: a life-threatening condition.	The SHR-1210 infusion was stopped immediately; the intravenous saline infusion was started. Bronchodilator is recommended, subcutaneous 1:1000 adrenaline solution 0.2-1 mg or 0.1-0.25 mg of 1:10000 adrenaline solution slowly intravenous injection. If necessary, receive intravenous diphenhydramine 50 mg and methylprednisolone 100 mg (or equivalent dose). Follow the institutional medical practices and guidelines for the treatment of anaphylaxis. Bedside was observed and closely monitored until recovery.	Termination of medication

Appendix 3 Tigiol dose based on body surface area

**100% dose level twice daily**		Number of pills	
		morning	night
body surface area (m^2)	Total dose (mg) *	20mg	20 mg
< 1.25	40	2	2
1.25 – 1.50	50	2	3
>1.5	60	3	3
**1 dose level twice daily**		Number of pills	
		morning	morning
body surface area (m^2)	Total dose (mg) *	20mg	20 mg
< 1.25	—	—	—
1.25 – 1.50	40	2	2

>1.5	50	2	3
**2 dose levels twice daily**		Number of pills	
		morning	morning
body surface area (m²)	Total dose (mg) *	20mg	20 mg
< 1.25	—	—	—
1.25 – 1.50	—	—	—
>1.5	40	2	2

Appendix 4, ECOG PS Score

state	And the ECOG physical fitness status score
0	Activity was completely normal without any difference from mobility before onset
1	Ability to walk freely and engage in light physical activity, including general housework or office work, but ability not to engage in heavy physical activity
2	He can walk freely and take care of himself, but has lost the ability to work and can get up no less than half of the day
3	Life can only take part of themselves, more than half of the day in bed or wheelchair
4	Bed and unable to take care of themselves

Appendix 5 Standardized operation procedure for postoperative intraperitoneal chemotherapy port placement for gastric cancer

Postoperative catheterization: After endoscopic exploration, the catheter was placed in the right lower abdomen, and the catheter was placed into the pelvic floor.

Drug selection and dose: albumin paclitaxel, dose of 80mg / m2, d1,21 days, 1 cycle, 4 cycles.

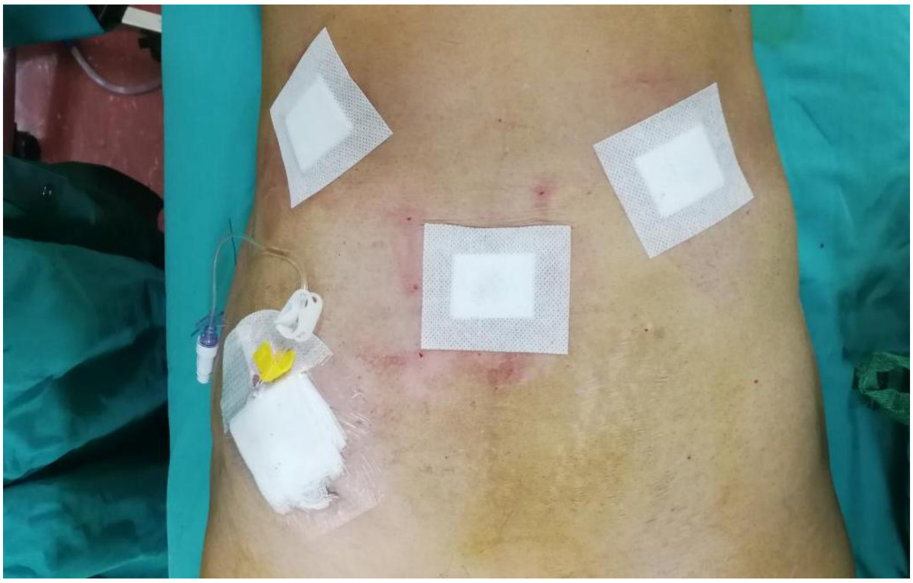

Monitoring during infusion: mainly monitoring blood pressure, pulse, body temperature, respiration, blood oxygen saturation, etc.;

Side effects and complications:

Delay exhaust for 1-3 days;

Adverse reactions of chemotherapy drugs, if occurred, should be treated routinely and symptomatic.
